# Acute renal failure in an AIDS patient on tenofovir: a case report

**DOI:** 10.1186/1752-1947-2-94

**Published:** 2008-03-31

**Authors:** Pinelopi P Kapitsinou, Naheed Ansari

**Affiliations:** 1 Department of Medicine, Jacobi Medical Center, Albert Einstein College of Medicine, Bronx, New York, USA; 2 Division of Nephrology, Department of Medicine, Jacobi Medical Center, Albert Einstein College of Medicine, Bronx, New York, USA

## Abstract

**Introduction:**

Tenofovir is a potent nucleotide analogue reverse-transcriptase inhibitor used with other antiretroviral agents for the treatment of human immunodeficiency virus (HIV) infection. Despite the absence of renal toxicity observed in the major clinical trials of tenofovir, several case reports of acute renal failure (ARF) and proximal tubule dysfunction have been described.

**Case presentation:**

We report a patient who developed ARF and Fanconi syndrome during treatment with tenofovir. Despite severe metabolic acidosis associated with a creatinine of 9.8 mg/dL (866 μmol/L), this patient's condition improved on discontinuation of tenofovir treatment without requiring renal replacement therapy.

**Conclusion:**

Vigilant screening of kidney function is required regularly after initiation of tenofovir due to possible appearance of renal failure.

## Introduction

Tenofovir is a nucleotide reverse-transcriptase inhibitor which was approved for use by the Food and Drug Administration in 2001 for the treatment of HIV. It belongs to the same class as adefovir and cidofovir which have well documented renal toxicities including proximal renal tubule cell dysfunction and acute renal failure (ARF) [1,2]. The described mechanism of tubular toxicity for the latter two drugs is cellular accumulation through increased entry from the hOAT (organic anion transporters located on the basolateral side of the tubule) and decreased efflux into tubular lumen mediated by the MRP 2 (Multidrug-Resistance-Protein) [3]. Similar effects were not expected with tenofovir due to decreased interaction with human organic transporter 1 and minimal mitochondrial toxicity in vitro [2,3].

Twenty seven cases of tenofovir related tubular dysfunction and Fanconi syndrome have been described in the medical literature. We describe another case of a patient in whom ARF and Fanconi syndrome developed during treatment with tenofovir.

## Case presentation

A 53-year-old woman with AIDS of 6 years duration developed progressive weakness, dyspnea on exertion and constipation. Her symptoms also included decreased appetite, weight loss and episodes of lightheadness. She had a history of drug and alcohol addiction, seizure disorder, stroke, pancreatitis and chronic low back pain and she was known to have been Hepatitis B and C positive since 2002. Antiretroviral therapy, consisting of abacavir, lamivudine and zidovudine, had been started in March 2002, when she was found to have Pneumocystis jirovecii pneumonia. She had not developed any other opportunistic infections. Eighteen months later, in October 2003, her HAART regimen was switched to tenofovir (300 mg/day), sustiva (600 mg/day) and Epivir (300 mg/day). At that time, her creatinine was 0.8 mg/dL (71 μmol/L). A recheck in December 2005 revealed a creatinine of 0.9 mg/dL (80 μmol/L) corresponding to eGFR 75 ml/min. She had been on this regimen without any change in the dose of tenofovir until she presented to hospital. Her other medications included aspirin 81 mg/day, folic acid and hydroxyzine. She had also been started on trimethoprime-sulfamethoxazole but discontinued this herself in April 2006.

On admission, clinical examination revealed signs of mild dehydration. Laboratory tests disclosed the following concentrations: sodium, 134 mEq/L; potassium, 3.4 mEql/L; chloride, 115 mEq/L; bicarbonate, 8 mEq/L; BUN, 57 mg/dL (20 mmol/L); creatinine, 9.8 mg/dL (866 mmol/L); phosphorous, 5.7 mg/dL (1.8 mmol/L); CPK, 119 U/L; uric acid, 4.9 mg/dL; lactate, 0.63 mmol/L and albumin 3.8 g/dL (38 g/L). Arterial blood gas showed academia (pH: 7.15) with appropriate respiratory response (pCO2 21 mmHg). In a urine sample, sodium was 44 mEq/L with a FeNa of 4%, potassium 39 mEq/L, chloride 43 mEq/L and creatinine 82 mg/dL (7249 mmol/L). Urinalysis showed marked glucosuria (294 mg/dL) with normoglycemia and proteinuria (124 mg/dL) and an absence of active urinary sediment. Urine pH was 6.0. The rates of fractional excretion of phosphorus and uric acid were 58% and 37% respectively. The findings of renal ultrasound were normal, as were the findings for all serologic tests. Her CD4+ lymphocyte count was 241 and her viral load 460 HIV RNA copies/ml.

Tenofovir therapy was discontinued and her HIV regimen was adjusted to abacavir, sustiva and epivir. Intravenous bicarbonate therapy was initiated with simultaneous potassium supplementation. Within the next few days, there was slow improvement in serum creatinine and bicarbonate levels but hypokalemia (minimum 2.5 mEq/L) recurred, requiring discontinuation of bicarbonate. Five days after her admission to our hospital, the patient discharged herself against medical advice. At that time she still had hypokalemia (2.7 mEql/L), low bicarbonate level (16 mEq/L) and a creatinine of 6.1 mg/dl (539 mmol/L). At follow-up at 7 months, her kidney function had returned to normal.

## Discussion

In short-term clinical trials, tenofovir did not exhibit more frequent nephrotoxicity compared to placebo [4]. Recently, however several case reports documenting nephrotoxicity have been described in the literature [5-8]. A number of different manifestations of kidney disease have been reported with tenofovir, including ARF, rhabdomyolysis, Fanconi syndrome and diabetes insipidus [6].

Many patients diagnosed with AIDS develop acute or chronic diarrheal syndromes with associated non-anion gap metabolic acidosis from bicarbonate loss in the stool. However, our patient did not report any episodes of diarrhea and the positive urine anion gap was not consistent with that possibility. Exposure to trimethoprime-sulfamethoxazole can induce acute interstitial nephritis. High-dose trimethoprime-sulfamethoxazole has been associated with unexplained RTA in a series of patients. The discontinuation of trimethoprime-sulfamethoxazole three months prior to presentation makes both of these scenarios in our case unlikely. Type B lactic acidosis resulting from mitochondrial dysfunction is well described for nucleoside reverse transcriptase inhibitors. No hyperlactemia was found in this patient. Other potential causes of severe metabolic acidosis were not identified. Our patient had typical findings of Fanconi syndrome (hyperchloremic metabolic acidocis, hypokalemia, glucosuria) but she was hyperphosphatemic. Hyperphosphatemia was attributed to decreased GFR whereas the possibility of pseudohyperphosphatemia secondary to multiple myeloma was ruled out with negative SPEP (serum protein electrophoresis). The constellation of ARF and Fanconi syndrome and the close temporal relationship between discontinuation of tenofovir and improvement of renal function suggest that tenofovir-induced nephrotoxicity was the most likely diagnosis. The creatinine of 9.8 mg/dL (866 mmol/L) and bicarbonate of 8 mEq/L are the highest and lowest respectively reported in the literature among the non-hemodialysis requiring cases of ARF secondary to tenofovir. The first case of hemodialysis requiring ARF secondary to tenofovir was of a 40 year-old HIV man who presented with oliguria, acidemia (pH 7.10, HCO3 6 mEql/L), lactate 7 mmol/L and creatinine of 20 mg/dL (1768 mmol/L)[7]. However, this patient was also receiving metformin, which could be implicated particularly in the setting of high lactate levels. The second case of hemodialysis in ARF induced by tenofovir was a 65 year-old-man with diabetes and AIDS who was admitted with creatinine, 7.1 mg/dL (628 mmol/L, GFR 6.8 ml/min), blood urea nitrogen, 68 mg/dL (24 mmol/L) and bicarbonate, 10 mEq/L [7]. This patient received two hemodialysis treatments for azotemia. Expecting recovery of kidney function after discontinuation of tenofovir treatment, we did not dialyse our patient, as she was asymptomatic.

In a recent review, Zimmermann et al. analyzed the findings for the 27 patients described in the literature with tenofovir-associated ARF since December 2002 [8]. The mean age was 45.5 years, with a ratio of men to women 3.5:1. The mean duration of tenofovir treatment was 11 months (range, 1–29 months). Our patient was taking tenofovir for 32 months, which is to our knowledge the latest presentation of tenofovir- induced ARF.

There are no known predictors of which patients will develop ARF associated with tenofovir. There was no correlation of CD4 cell count and plasma HIV load with the development of ARF [8]. However, low baseline GFR may contribute to tenofovir's toxicity via increase in the serum concentration [8].

Similarly, no predictors for occurrence of Fanconi syndrome exist. A recent study identified genetic variants of hOAT1 and investigated potential effects on the functional properties of this transporter. Kinetic analysis indicated that the transport affinity for the nucleoside phosphonate analogues adefovir, cidofovir and tenofovir seemed to be decreased in the R50H-hOAT1 variant compared with the wild type. This study raises the genetic variation in hOAT1 as potential explanation of "different handling" of these drugs with the associated clinical implications [9].

Tenofovir is primarily excreted by the kidneys. Elimination is accomplished by glomerular filtration as well as by active tubular secretion. There are several medications that compete for renal tubular secretion, including acyclovir, cidofovir, valcyclovir, ganciclovir and valganciclovir. Co-administration may lead to increased serum levels of tenofovir. Additionally, tenofovir causes CYP1A2 inhibition, and drug levels may be increased when given with other antiretroviral medication. Administration of ritonavir alone or with lopinavir has been shown to increase the maximum serum concentrations of tenofovir by 30%, while didanosine and atazanavir also have been described to have potential interactions with tenofovir [10].

Notably, our patient was not receiving tenofovir concurrently with any of the above listed medications.

Until recently, no long-term renal impairment was expected as a consequence of tenofovir-related nephrotoxicity. However the incomplete recovery of kidney function in 5 out of 27 reported cases after a mean duration of follow-up of 7.5 months raises serious concerns for occurrence of chronic kidney disease after discontinuation of tenofovir [8].

A follow-up of serum creatinine, urinalysis and electrolytes should be performed in patients taking tenofovir. Early diagnosis is important so that this medication can be discontinued in a timely manner and life-threatening electrolyte imbalances can be avoided. Hemodialysis will not be necessary in the majority of cases, given the rapid resolution of ARF with the discontinuation of tenofovir. Physicians should continue to be vigilant in screening patients well after initiation of tenofovir due to possible late appearance of renal failure. In cases of ARF occurrence, persistence of kidney damage should be considered as a possibility so that early optimization of coexistent risk factors can be attempted.

## Conclusion

We describe the case of a patient in whom ARF and Fanconi syndrome developed during treatment with tenofovir. Vigilant screening of kidney function is required regularly after initiation of tenofovir due to possible late appearance of renal failure.

## Competing interests

The author(s) declare that they have no competing interests.

## Authors' contributions

PK has been involved in the conception, design, drafting, and revising the manuscript. NA has been involved in the diagnosis and treatment of the patient and revising the manuscript.

## Consent

Written informed consent was obtained from the patient for publication of this case report and accompanying images. A copy of the written consent is available for review by the Editor-in-Chief of this journal.
